# Improvement in Flavonoids and Phenolic Acids Production and Pharmaceutical Quality of Sweet Basil (*Ocimum basilicum* L.) by Ultraviolet-B Irradiation

**DOI:** 10.3390/molecules21091203

**Published:** 2016-09-09

**Authors:** Ali Ghasemzadeh, Sadegh Ashkani, Ali Baghdadi, Alireza Pazoki, Hawa Z. E. Jaafar, Asmah Rahmat

**Affiliations:** 1 Department of Crop Science, Faculty of Agriculture, Universiti Putra Malaysia, 43400 Serdang, Selangor, Malaysia; ali_baghdadi@yahoo.com (A.B.); hawazej@upm.edu.my (H.Z.E.J.); 2 Department of Agronomy and Plant Breeding, Yadegar-e-Imam Khomeini (RAH) Shahre Rey Branch, Islamic Azad University, Tehran, Iran; ashkani.sadegh@gmail.com (S.A.); pazoki_agri@yahoo.com (A.P.); 3 Department of Nutrition and Dietetics, Faculty of Medicine and Health Sciences, Universiti Putra Malaysia, 43400 Serdang, Selangor, Malaysia; profasmah@gmail.com

**Keywords:** *Ocimum basilicum* L., UV-B, TFC, TPC, CHS activity, antioxidant activity, antiproliferative activity

## Abstract

Sweet basil (*Ocimum basilicum* Linnaeus) is aromatic herb that has been utilized in traditional medicine. To improve the phytochemical constituents and pharmaceutical quality of sweet basil leaves, ultraviolet (UV)-B irradiation at different intensities (2.30, 3.60, and 4.80 W/m^2^) and durations (4, 6, 8, and 10-h) was applied at the post-harvest stage. Total flavonoid content (TFC) and total phenolic content (TPC) were measured using spectrophotometric method, and individual flavonoids and phenolic acids were identified using ultra-high performance liquid chromatography. As a key enzyme for the metabolism of flavonoids, chalcone synthase (CHS) activity, was measured using a CHS assay. Antioxidant activity and antiproliferative activity of extracts against a breast cancer cell line (MCF-7) were evaluated using 1,1-diphenyl-2-picrylhydrazyl (DPPH) assays and MTT (3-(4,5-dimethylthiazol-2-yl)-2,5-diphenyltetrazolium bromide) assays, respectively. UV-B irradiation at an intensity of 3.60 W/m^2^ increased TFC approximately 0.85-fold and also increased quercetin (0.41-fold), catechin (0.85-fold), kaempferol (0.65-fold) rutin (0.68-fold) and luteolin (1.00-fold) content. The highest TPC and individual phenolic acid (gallic acid, cinnamic acid and ferulic acid) was observed in the 3.60 W/m^2^ of UV-B treatment. Cinnamic acid and luteolin were not detected in the control plants, production being induced by UV-B irradiation. Production of these secondary metabolites was also significantly influenced by the duration of UV-B irradiation. Irradiation for 8-h led to higher TFC, TPC and individual flavonoids and phenolic acids than for the other durations (4, 8, and 10-h) except for cinnamic acid, which was detected at higher concentration when irradiated for 6-h. Irradiation for 10-h significantly decreased the secondary metabolite production in sweet basil leaves. CHS activity was induced by UV-B irradiation and highest activity was observed at 3.60 W/m^2^ of UV-B irradiation. UV-B treated leaves presented the highest DPPH activity and antiproliferative activity with a half-maximal inhibitory concentration (IC_50_) value of 56.0 and 40.8 µg/mL, respectively, over that of the control plants (78.0 and 58.2 µg/mL, respectively). These observations suggest that post-harvest irradiation with UV-B can be considered a promising technique to improve the healthy–nutritional and pharmaceutical properties of sweet basil leaves.

## 1. Introduction

Flavonoids, which are important secondary metabolites in plants, are involved in resisting environmental stress and regulating the growth of plants [1]. Abundant in leaves, flowers, fruits, and roots, they play important roles in herbal medicines and healthy food products. The biosynthesis of flavonoids in plants begins with the shikimate pathway, and intermediate products, such as chalcone and isoflavonoids, are formed via the phenylpropanoid pathway [2,3]. These flavonoids are then conjugated to other flavonoids and glycose to form final products including anthocyanin and flavonol glycoside. However, the enzymes in these biosynthesis pathways (CHS and phenylalanine ammonia-lyase) are affected by many environmental factors including light, temperature, and concentration of CO_2_, soil nutrients, and water [4,5,6]. Nascimento et al. [7] showed that ultraviolet (UV) light greatly influences phytochemical and antioxidant activities in *Kalanchoe pinnata*. UV-B irradiation has been recommended by several studies as a mean to enhance the nutritional quality of fruits and crops [8,9,10]. The DNA and tissues of plants can be injured by UV-B irradiation [11]. High levels of UV irradiation, lead to increased concentrations of antioxidants in plants and is considered as a tool for enhancing contents of bioactive compounds in fresh crops, herbs, fruit and vegetables after harvest [12,13]. Therefore, to find an optimized condition that increases flavonoids primarily in the leaves, there have been increased research studies on exposing plants to UV-B irradiation at different temperatures or air components, as well as comparing the effects owing to the age of the leaves. It has been reported that irradiating parsley (*Petroselinum hortense*) with UV-B over certain durations causes a 4-fold increase in the quantity of flavonoids [14]. Furthermore, Sun et al. [15] showed that for three different ages of *Ginkgo biloba* leaves, significant differences in the changes of the amount of flavonoids after UV-B irradiation were obtained. Moreover, Harbaum-Piayda et al. [16] indicated that the flavonoid content of pakchoi (*Brassica campestris*) increased significantly after UV-B irradiation under different temperature conditions. Thus, the optimized condition of UV-B irradiation to increase flavonoids differs between species owing to variations in wax content, tissue thickness, enzyme activity, and photoreceptors in the leaves.

Sweet basil (*Ocimum basilicum* Linnaeus) is an aromatic herb that grows to 20–80 cm height. The stem is glabrous and woody at the base and leaves are large, green in colour, broadly epitical, 2.5–5 cm × 1–2.5 cm in size. Flowers are small (3 mm), red, pink or white in colour and arranged in terminal spikea [17]. Sweet basil is a highly useful plant because the whole plant has been utilized in traditional medicine from antiquity in the form of household remedies against various human ailments. It is a popular herb, valued for its rich and spicy, mildly peppery flavor with a trace of mint and clove, and has been used widely for flavoring confectionary as well as other food products. Today, healthy food products are prepared from sweet basil leaves and are commonly utilized in traditional medicine to relieve stress, prevent gout, and for other health benefits [18]. Recently, many researchers have reported that there are various kinds of flavonoids in sweet basil leaves that have antioxidant [18], anti-inflammatory [19], anticancer [20], antimicrobial [21], and wound-healing [22] activities. Phytochemical constituents of sweet basil has been investigated by several studies and by now more than 200 phytochemical compounds like as monoterpenes, limonene, myrcene, terpinolene, flavonoids (quercetin, kaempferol, rutin), phenolic acids (*p*-coumaric acid, caffeic acid, caftaric acid), steroids and vitamins (A, C, E, K) have been identified in this herb [23]. Therefore, the possibility of using simple methods to increase flavonoid content in sweet basil leaves, to enhance the bioactivities in the leaf extract simultaneously, is worthy of further investigation. The majority of studies have utilized UV-B irradiation as a pre-harvest treatment while the plants were growing; however, post-harvest treatment might be more efficient (pre-harvest treatment of UV-B decrease photosynthetic rate and plant growth). The aim of the present study is to evaluate the effect of UV-B irradiation on the phytochemical constituents of sweet basil leaves and its antioxidant and antiproliferative activities. In addition, owing to the various sensitivities to UV-B irradiation in different species, the dosage effects of certain factors (irradiation intensity and irradiation duration) for approaching an optimal condition were studied.

## 2. Results and Discussion

### 2.1. Changes in TFC and TPC under Different UV-B Irradiation Intensities

The changes in total flavonoid content (TFC) and TPC in the leaves are shown in Table 1. Each irradiation intensity (2.30, 3.60, and 4.80 W/m^2^) applied for a duration of 6-h resulted in a significant increase in TFC, especially in the 2.30 and 3.60 W/m^2^ treatments. Irradiating at the 3.60 W/m^2^ intensity resulted in a 0.76-fold increase in TFC from 22.26 mg QE/g DM to 39.18 mg QE/g DM. However, the higher irradiation intensity (4.80 W/m^2^) did not demonstrate a higher level of TFC, indicating that when the intensity is too high, the amount of TFC consumed, which captures the reactive oxygen species (ROS) and repairs the oxidative damage caused by the UV-B irradiation, was almost equal to the increase of TFC [8]. Thus, if the irradiation intensity is higher than 4.80 W/m^2^, the amount of TFC may actually decrease. The increase of TFC, showed significant difference between the 2.30 and 3.60 W/m^2^ intensities, indicating that both these irradiation intensities are in a suitable range to increase the TFC. However, controlling the 2.30 W/m^2^ intensity required more distance between the lamps and leaves than for the 3.60 W/m^2^ intensity, and the longer distance increased the amount of space needed, which might make the post-treatment more inconvenient. Therefore, 3.60 W/m^2^ was the most appropriate intensity to implement to increase the amount of TFC.

On the other hand, after irradiation with different intensities of UV-B, the quantities of TPC in sweet basil leaves increased from 48.22 mg GAE/g DM in the control plants to 50.41 mg GAE/g DM in 2.30 W/m^2^ intensity, 56.12 mg GAE/g DM (3.60 W/m^2^), and decreased to 49.16 mg GAE/g DM when exposed to 4.80 W/m^2^ of UV-B irradiation (Table 1). Our findings are consistent with Schreiner et al. [10] who discovered that TPC in *Tropaeol majus* leaves increased after irradiation with UV-B. Moreover, the results in Table 1 show that TPC increased further when irradiation intensities of UV-B shifted from 2.30 W/m^2^ to 3.60 W/m^2^. Thus, there appears to be a correlation between irradiation intensities of UV-B and TPC. Eichholz et al. [9] indicated that UV-B irradiation could enhance phenylalanine ammonia-lyase and peroxidase activities to promote the derivatization and polymerization of phenolic compounds such as flavonoids. In addition, plants produce higher amounts of ROS as signals after UV-B irradiation, and ROS can be quenched by phenolic compounds [24]. Hence, after irradiation, TPC in sweet basil leaves might be the precursor for flavonoid biosynthesis and be decreased in other pathways, such as the reactions with peroxidase and ROS when the intensity of UV-B irradiation was too high.

Several identification and separation techniques have previously been reported in order to identify secondary metabolites from plant materials [25,26,27]. In the present study, three phenolic acids (gallic acid, cinnamic acid, and ferulic acid) and five flavonoids (quercetin, catechin, kaempferol, rutin and luteolin) were identified in the sweet basil leaf extracts using ultra-high performance liquid chromatography (Table 1). With increasing UV-B irradiation from 2.30 to 4.80 W/m^2^, the amount of gallic acid and ferulic acid increased significantly in sweet basil leaves compared to control plants. More interestingly, cinnamic acid was not detected in the control plant extracts, but was detected in the leaves of plants exposed to UV-B radiation. Similar to gallic acid and ferulic acid, with increasing UV-B radiation intensity from 2.30 to 4.80 W/m^2^ the amount of cinnamic acid increased significantly from 3.24 mg/g DM to 3.91 mg/g DM. More interestingly, ferulic acid was not detected at higher UV-B irradiation (4.80 W/m^2^). Individual flavonoids showed the same results as the phenolic acids. Increasing UV-B irradiation intensity from 2.30 to 4.80 W/m^2^ resulted in an increase in the amount of quercetin, catechin, and kaempferol in the leaf extracts than that in the control plants. The highest content of quercetin (3.87 mg/g DM), catechin (4.0 mg/g DM), kaempferol (2.90 mg/g DM) and rutin (1.96 mg/g DM) was obtained from leaf extracts exposed to 3.60 W/m^2^ of UV-B irradiation. Luteolin was not detected from control plants and its synthesis was induced by UV-B irradiation. The highest content of luteolin (0.88 mg/g DM) was observed at 2.30 W/m^2^ of UV-B irradiation but there is no significant differences between 2.30 and 3.60 W/m^2^ of UV-B irradiation for luteolin content. With increasing UV-B irradiation from 3.60 to 4.80 W/m^2^, the amount of flavonoid compounds decreased significantly (*p* < 0.05).

Although the intensity of UV-B irradiation affects flavonoids of sweet basil leaves, the mechanisms of flavonoid biosynthesis differ between species. For example, Olsson et al. [28], who assessed the effects of UV-B irradiation on *Brassica napus*, reported that quercetin glucoside content increased to a greater extent than did the kaempferol glucoside content, revealing that the quercetin-type flavonoids increased more than the kaempferol-type flavonoids. However, the increased ratio of quercetin in *Ginkgo biloba* leaves was the same as that of kaempferol in the research of Sun et al. [15]. Overall, these studies showed various patterns in the biosynthesis of diverse kinds of flavonoids between different species. However, the reasons for these different patterns have not been clearly determined, but it is important to understand these patterns for different applications of various species. Hence, the different mechanisms of biosynthesis between species of plants and structure of flavonoids are worthy of continued investigation.

### 2.2. Changes of TFC and TPC under Different UV-B Irradiation Durations

To understand the effects of UV-B irradiation duration on TFC and TPC, sweet basil leaves were irradiated under identical conditions of 25 °C temperature, 90% relative humidity, and 3.60 W/m^2^ irradiation intensity, with various irradiation durations (4, 6, 8, and 10-h). The results showed that TFC increased significantly (*p* < 0.05) after the 4–8 h UV-B irradiation durations (Table 2). After the 4-h irradiation, TFC increased from 20.88 mg QE/g DM to 37.76 mg QE/g DM, and following 6-h irradiation, TFC increased to 40.11 mg QE/g DM. After 8-h irradiation, TFC increased to 41.19 mg QE/g DM; however, this increase was not significant compared to 6-h irradiation. In contrast, following 10-h irradiation, TFC decreased significantly to 28.11 mg QE/g DM, which was lower than the value after 4-h irradiation. Sun et al. [15] reported TFC of approximately a 1.56-fold increase in mature leaves of *G. biloba* after 2-h irradiation and a 1.57-fold increase after 4-h irradiation. These results indicate that irradiation for specific durations might lead to the maximum increase in the ratio of TFC. Because leaf tissues might be damaged by an overdose of UV-B irradiation, the TFC would be rapidly consumed to protect the leaves [29,30]. In addition, there was the highest threshold for UV-B irradiation to increase TFC, with the increasing ratio of TFC decreased after longer irradiation durations. Therefore, applying moderate irradiation durations is the most efficient method to increase TFC. According to these results, both 6-h and 8-h irradiation durations were ideal for increasing the TFC of sweet basil leaves. However, when considering the cost of longer irradiation duration, the 6-h irradiation duration was more efficient, and so it was chosen for subsequent experiments. As can be seen in Table 2, individual flavonoid content increased with the increase in irradiation time from 4 to 8-h; however, after 8 to 10-h irradiation, this decreased significantly. The highest content of quercetin (3.85 mg/g DM), catechin (4.67 mg/g DM), kaempferol (3.80 mg/g DM), rutin (2.12 mg/g DM) and luteolin (0.96 mg/g DM) were observed at 8 h of UV-B irradiation. For luteolin synthesis no significant differences was observed between 6 and 8-h. UV-B radiation induces the synthesis of key enzymes of the phenylpropanoid pathway; thus, under enhanced UV-B radiation, plants usually increase de novo synthesis of flavonoids [31,32].

The results from the present study reveal that TPC increased significantly with increasing irradiation time up to 8-h (Table 2). The lowest TPC was observed in the non-treated plants, with TPC increasing from 44.52 mg GAE/g DM in the control plants to 47.53 mg GAE/g DM (4-h), 51.12 mg GAE/g DM (6-h) and 55.79 mg GAE/g DM (8-h). Continuation of irradiation to 10-h decreased TPC significantly to 43.20 mg GAE/g DM, which was lower than control plants. The content of gallic acid, cinnamic acid and ferulic acid in the sweet basil leaves increased significantly with increasing irradiation time. The content of cinnamic acid in sweet basil leaves increased until the 6-h irradiation point and then decreased until the 10-h irradiation time. The highest content of gallic acid (6.07 mg/g DM) and ferulic acid (5.10 mg/g DM) was observed at 8-h irradiation, while the highest content of cinnamic acid (3.96 mg/g DM) was observed under the 6-h irradiation duration. After UV-B irradiation, studies have shown that there is an increase in RNA transcription level for enzymes participating in the flavonoid biosynthesis of plants, such as phenylalanine ammonia-lyase, cinnamic acid 4-hydroxylase (C4H), 4-coumarate-CoA ligase (4CL), chalcone synthase (CHS), and flavone synthase (FNS), and their increasing ratios and stimulation durations are not the same [33,34]. In RNA transcription of CHS, which is more directly related to flavonoid biosynthesis, this can be sustained at high levels for a long duration [15]. According to these previous studies, it could be hypothesized that during the long irradiation duration and reaction time, the induced flavonoid biosynthesis lasted longer than that of upstream phenol biosynthesis. Therefore, phenolic compounds, which are precursors of flavonoid biosynthesis, were consumed quickly to form flavonoids. In general, irradiation up to 8-h resulted in improving and enhancing of secondary metabolites in sweet basil, but, continuing of irradiation to 10-h resulted in decreasing of secondary metabolites. The results of our study was consistent with Scattino et al. [35] who reported that 12-h irradiation with UV-B resulted in increasing of flavonoids in peach skin, while when irradiation time increased to 24-h flavonoids content decreased significantly. Based on obtained results from phytochemical analysis, 3.60 W/m^2^ UV-B irradiation for duration of 8-h (with highest flavonoid and phenolic acid contents) and non-treated leaves (as a control) were chosen for evaluation of antioxidant and antiproliferative activities. 

### 2.3. Chalcone Synthase (CHS, EC 2.3.1.74) Activity

**Figure 1:**
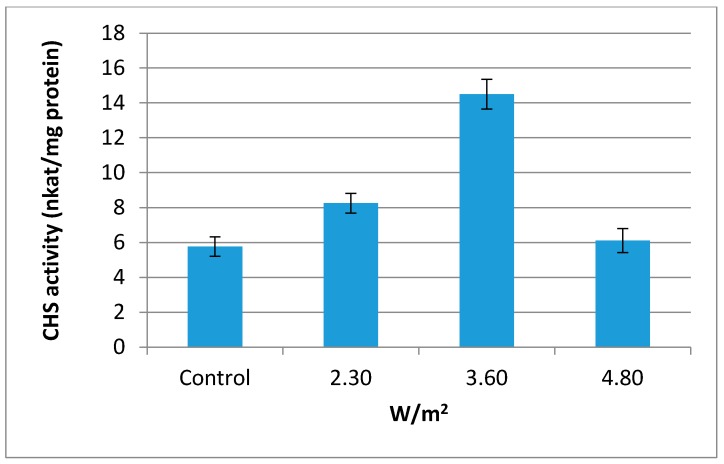
Effect of UV-B irradiation (3.60 W/m^2^, 8-h) on CHS enzyme activity in sweet basil leaves.

Enzyme chalcone synthase (CHS, EC 2.3.1.74) has been discovered and reported as a key enzyme for the metabolism of flavonoid in plant cells [36]. In this study, CHS activity in sweet basil leaves was influenced significantly by UV-B irradiation. As can be seen from Figure 1, highest CHS activity was observed at 3.60 W/m^2^ followed by 2.30 and 4.80 W/m^2^. Control plants represent lowest CHS activity. Induction of CHS enzyme activity by UV-B irradiation was reported by previous studies [37,38,39]. Flavonoids are derived from 4-coumaroyl-CoA and malonyl-CoA in the presence of CHS. This indicates that CHS is an important enzyme in flavonoid synthesis. According to the current results, it is hypothesized that the increment of polyphenolic compounds in the sweet basil leaves at 3.60 W/m^2^ could be attributed to an increase in CHS activity.

### 2.4. Antioxidant Activity 

**Figure 2:**
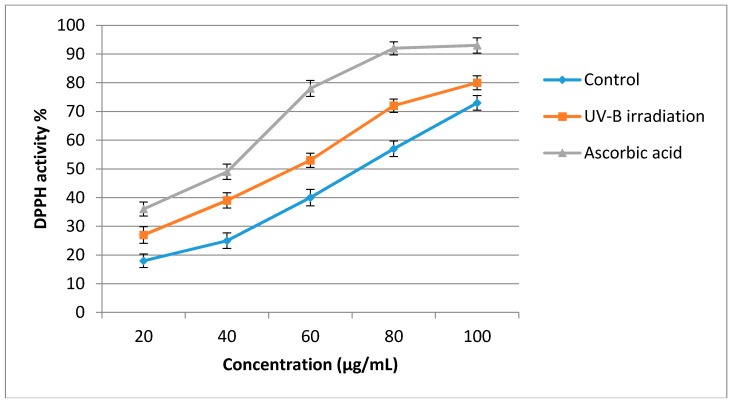
Antioxidant activity of sweet basil non-treated leaves and UV-B treated (3.60 W/m^2^, 8-h) leaves using DPPH assay. Bars represent standard errors of the means.

The results of the assessment of antioxidant activity using the DPPH assay are shown in Figure 2. According to these results, the antioxidant activity of sweet basil leaf extracts was significantly enhanced after using UV-B irradiation treatment (3.60 W/m^2^, 8-h) over that of the extracts from the control plants. The DPPH radical scavenging activity of sweet basil was significantly increased (*p* < 0.05) as sample concentration increased. The half-maximal inhibitory concentration (IC_50_) value of UV-B treated plants was 56 µg/mL while the IC_50_ of the control plants was 78 µg/mL and ascorbic acid has an IC_50_ of 41 µg/mL. Lower IC_50_ values represent stronger free radical inhibition and strong free-radical inhibitors are active at low concentrations. From the results of the present study, after the application of UV-B irradiation, the antioxidant activity of the sweet basil leaves significantly increased. This increase could be related to an increase in secondary metabolites such as flavonoids and phenolic acids in UV-B treated plants. Positive correlation between secondary metabolites content and antioxidant activity of herbs has been recorded in previous studies [40,41,42,43]. In a more recent study, it was reported that the antioxidant activity of tomato fruit was significantly increased following the increase in secondary metabolites when treated with UV-B irradiation [44]. The results obtained from the present study are consistent with those obtained by Rybarczyk-Plonska et al. [45] and Harbaum-Piayda et al. [46], who reported the induction of secondary metabolites production and enhancement of antioxidant activity in broccoli flower and cabbage leaves, respectively, after UV-B irradiation. Antioxidant activities of 23 varieties of Iranian basil have also been investigated and the results showed that there is positive linear relationship between the total phenolic content and antioxidant activity in all varieties [47].

### 2.5. Antiproliferative Activity 

**Figure 3:**
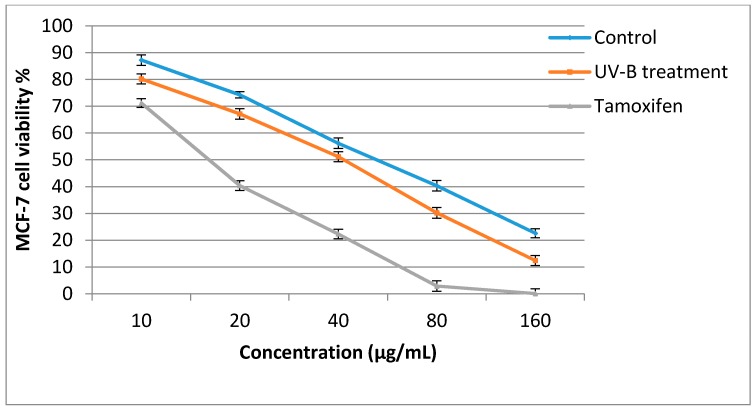
Effect of sweet basil non-treated leaves and UV-B treated (3.60 W/m^2^, 8-h) leaves against breast cancer cell line (MCF-7) viability. Bars represent standard errors of the means.

**Figure 4:**
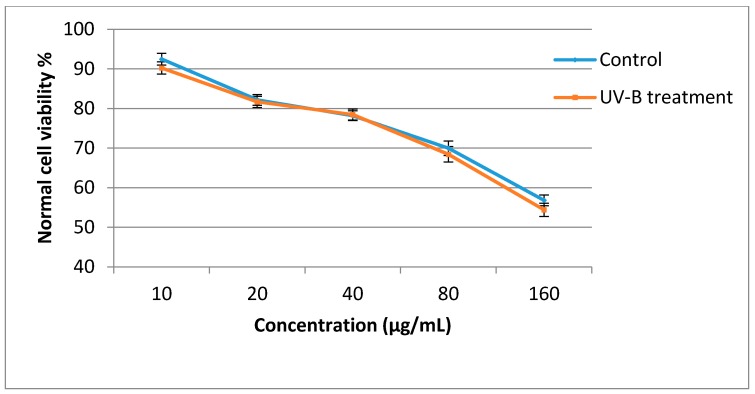
Effect of sweet basil non-treated leaves and UV-B treated (3.60 W/m^2^, 8-h) leaves against normal cell (MCF-10A) viability. Bars represent standard errors of the means.

The antiproliferative activities of the sweet basil leaf extracts against the MCF-7 cell line were significantly influenced by UV-B treatment (Figure 3). In the control plants, when plant extract concentration was increased from 10 to 160 µg/mL, the cancer cell viability decreased from 87.24% to 22.6%, while in UV-B treated plants the cancer cell viability decreased from 80.20% to 12.40%. The antiproliferative activity of sweet basil leaf extracts in the control and UV-B treated plants was lower than that of Tamoxifen, which was used as a positive control. The IC_50_ value of UV-B treated plants against the MCF-7 cell line was 40.8 µg/mL, while that of control plants was 58.2 µg/mL, and the sweet basil leaves also had higher IC_50_ value than did tamoxifen (IC_50_ = 17.9 µg/mL). When the leaf extract concentration was increased from 10 to 160 µg/mL, normal cell viability decreased from 88.93% to 56.83% in the control plants and from 86.80% to 54.40% in the UV-B treated plants. At concentration of 40.8 µg/mL(IC_50_ of UV-B treated plants) 78.9% normal cell viability was observed while, at a concentration of 58.2 µg/mL(IC_50_ of non-treated plants) 76.2% normal cell viability was recorded (Figure 4). Previous studies have reported that the antiproliferative activity of herbs is associated with their phytochemical content [40,41,48]. Antiproliferative activity of sweet basil leaves against the human cervical cancer cell line (HeLa) with IC_50_ value of 164.6 µg/mL has been reported previously [49]. In a recent study, the cytotoxicity effect of sweet basil leaves were evaluated against HeLa and the human laryngeal epithelial carcinoma cell line, with the results showing that sweet basil represent a potent cytotoxicity effect with IC_50_ values of 90.5 and 96.3 µg/ mL, respectively [20]. Arshad et al. [50] reported antiproliferative activity of sweet basil extract against MCF-7 cells with IC_50_ values of 71 µg/mL. These studies all tested the antiproliferative activities of normal sweet basil leaves and, to the best of our knowledge, there is no information regarding the antiproliferative activity of sweet basil leaves treated with UV-B irradiation. Therefore, the results of the present study are useful for future studies. 

### 2.6. Correlation Analysis 

It is important to evaluate the correlation between biological activity of herbs and secondary metabolites content in order to introduce corresponding compounds for biological activity. Knowing of this could help researchers establish suitable conditions or to use suitable techniques in order to enhance these highlighted compounds. In the current study, correlation analysis between phytochemicals and biological activities of sweet basil extracts was done for optimized UV-B irradiation condition (3.60 W/m^2^ and 8-h irradiation). Correlation coefficient analyses showed a significant relationship between phytochemical content and breast cancer proliferation. Antiproliferative and antioxidant activity of sweet basil mostly correlated with flavonoids followed by phenolic acids. A negative and significant correlation coefficient was also found between breast cancer proliferation and phytochemicals ranging from R^2^ = −0.7433 to −0.9327 (Table 3). Between identified phytochemicals, quercetin and kaempferol showed a strong negative correlation with MCF-7 (R^2^_Q_ = −0.9327; R^2^_K_ = −0.9240) cancer proliferation. In current study, positive and significant correlation was observed between phytochemicals and antioxidant activity ranging from R^2^ = 0.8039 to 0.9315. Between identified phytochemicals, quercetin (R^2^ = 0.9168) and kaempferol (R^2^ = 0.9315) more correlated with antioxidant activity. Several studies reported a significant correlation between the pharmaceutical activity of herbs and the phytochemical content [41,51]. Based on obtained results it seems that quercetin and kaempferol are responsible phytochemicals for antiproliferative and antioxidant activity of sweet basil.

## 3. Materials and Methods

### 3.1. Plant Samples 

Sweet basil seeds (genotype: EC778531, Research Institute of Forest and Rangeland, Tehran, Iran) were sterilized with 1% hypochlorite solution for 2–3 min then washed thoroughly with tap water. All seeds were sown into 77-cell trays containing a soil–sand–peat mixture (1:1:1 ratio, *v*/*v*). The seedlings at four weeks of age with three to four leaves were transplanted into 3 L plastic pots containing a coco peat–burnt rice husk mixture (1:1 ratio, *v*/*v*). A medium fertilizer dose of 40:40:40 kg/ha of N, P_2_O_5_, and K_2_O was added, as this is the recommended dose for economic yield. For irrigation, a super drip irrigation system was utilized. The mean daily temperature 30 ± 2 °C, mean relative humidity 75%–80% and highest irradiance level at 1480 μmol/m^2^/s and whilst minimum at 52 μmol/m^2^/s. Plants were harvested at four months, with leaves separated after harvesting and washed with tap water. 

### 3.2. UV-B Radiation 

The UV-B irradiation conditions followed the method reported by Sun et al. [15] with minor modifications. All leaves, placed side by side without overlap, were irradiated under UV-B in a plant growth chamber (GC-101H, Firstek, New Taipei, Taiwan) under fixed humidity (90%). After UV-B irradiation, the treated samples were stored for 24 h in darkness to complete the adaptation reaction. Compounds from these treated samples were then extracted using 95% ethanol for further analysis. To determine the optimal irradiation of UV-B necessary to increase flavonoid levels, groups of samples were irradiated separately using UV-B lamps (TL 40 W/12 RS, 280–320 nm, Philips, Philips, Amsterdam, Netherlands) with different irradiation intensities at approximately 2.30, 3.60, and 4.80 W/m^2^ at 25 °C based on preliminary experiments. In addition, samples were irradiated to the optimal irradiation intensity of UV-B for different irradiation durations (4, 6, 8, and 10-h).

### 3.3. Preparation of Plant Extracts

Fresh leaves were dried using a freeze dryer. Dried samples (5 g) were ground into powder followed by extraction with distilled water (100 mL). Solutions were refluxed for 2-h at 65 °C, then cooled and filtered through filter paper (No. 1, Whatman, Singapore) in a filter funnel, followed by evaporation under reduced pressure in an Eyela rotary evaporator (Miyagi, Japan) to remove excess solvent. Residue was freeze dried and dried extracts were kept at −20 °C for future analysis. 

### 3.4. Estimation of Total Phenolic Content (TPC)

Total phenolic content was determined using the spectrophotometric method according to the Folin-Ciocalteu assay. Crude extracts (0.25 mg) were dissolved in methanol (10 mL) and 200 µL of the solution was diluted in distilled water (20 mL). Folin-Ciocalteu reagent (diluted 10-fold; 1 mL) was added and the mixture was incubated in total darkness for 10 min at room temperature. After this time, sodium carbonate 7.5% (1 mL) was added and the mixture incubated for 30 min, then the absorbance of the solution was read at 765 nm using a spectrophotometer (UV2550, Shimadzu, Kyoto, Japan). Different concentrations of gallic acid (0.062, 0.125, 0.250, 0.500, and 1 mg/mL) were used to prepare the calibration curve. Results were expressed as milligrams of gallic acid equivalents (GAE)/g DM. 

### 3.5. Estimation of Total Flavonoid Contents (TFC)

Total flavonoid content of the crude extracts was determined by using the aluminium chloride spectrophotometric method. Crude extract (0.25 mg) was dissolved in methanol (10 mL). Extracts (1 mL) were mixed with NaNO_2_ solution (4 mL, 1:5, *w*/*v*) and incubated at room temperature for 6 min. After this time, AlCl_3_ solution (0.3 mL, 1:10, *w*/*v*) was added, the reagents were mixed well, and the reaction was allowed to stand for another 6 min. immediately after that, 1 M NaOH solution (2.0 mL) was added to each extract and incubated for 10 min at room temperature. The absorbance of the solutions was read at 510 nm using a spectrophotometer (UV2550, Shimadzu). Different concentrations (0.031, 0.062, 0.125, 0.250, 0.500 mg/mL) of quercetin standard were used to prepare a calibration curve. Results were expressed as milligram quercetin equivalents (QE)/g DM. Measurements were performed in triplicate and values are the average of three replicates.

### 3.6. Identification of Flavonoids and Phenolic Acids

Ultra-high performance liquid chromatography (UHPLC, 1290 Infinity Quaternary LC System, Agilent, Santa Clara, CA, USA) was used to separate and identify the phenolic acids. The chromatographic system conditions were set as follows: mobile phase, 0.03 M orthophosphoric acid (A) and methanol HPLC grade (B); detector, UV 280–360 nm; column, C18 column (5.0 µm, 4.6 mm inner diameter [ID] × 250 mm); column oven temperature, 35 °C; and flow rate, 1.0 mL/min. Gradient elution was performed as follows: 0–10 min, 10% B; 10–15 min, 50% B; 15–20 min, 100% B; and finally 5 min for washing. All standards (gallic acid, cinnamic acid, ferulic acid, quercetin, catechin, kaempferol, rutin and luteolin) were purchased from Sigma-Aldrich (M) Sdn Bhd, Selangor, Malaysia. Linear regression equations were calculated using Y = aX ± b, where X is the concentration of the related compound and Y the peak area of the compound obtained from UHPLC. The linearity was established by the coefficient of determination (R^2^). UHPLC analysis was performed in triplicate and values are the average of three replicates.

### 3.7. Chalcone Synthase (CHS) Assay

The CHS was extracted from 0.4 g of leaves with a solution of 1 mM 2-mercaptoethanol dissolved in 0.1 M borate buffer (1 mL, pH 8.8) at 4 °C. Subsequently, Dowex l × 4 resin (0.1 g) was added to the solution and the mixture rested for 10 min. The solution was then centrifuged at 15,000 rpm for 10 min to remove the resin. The supernatant was transferred to a tube, and Dowex resin (0.2 g) was added and the mixture left standing for 20 min. The resin was removed from solution after centrifugation at 15,000 rpm for 15 min. The supernatant (100 μL) was mixed gently with 10 mM potassium cyanide and following that Tris-HCI buffer (1.89 mL, pH 7.8) was added. Subsequently, chalcone (10 mg) was added to ethylene glycol monomethyl ether (10 μL), mixed with enzyme extract, and the reaction allowed to proceed for 1 min at 30 °C. The absorbance was measured at 370 nm.

### 3.8. In Vitro Evaluation of Antioxidant Activity

#### 1,1-Diphenyl-2-picrylhydrazyl (DPPH) Assay

The DPPH assay was used in order to evaluate the free radical scavenging activity of sweet basil extracts. DPPH was dissolved in methanol at a concentration of 100 µM. The DPPH solution (3 mL) was mixed with 3 mL of various concentrations of plant extracts and incubated in a dark room for 20 min at 27 °C. After incubation, the absorbance of the samples was read at 517 nm using a spectrophotometer (UV2550, Shimadzu). Ascorbic acid was used as a positive control. The scavenging activity was calculated using the following formula:

% inhibition = [(absorbance control − absorbance sample)/absorbance control)] × 100
(1)

### 3.9. Determination of AntiProliferative Activity

#### 3.9.1. Cell Culture and Treatment

Estrogen receptor (ER)-positive MCF-7 breast cancer cell and normal cell line (MCF-10A) were procured from the laboratory of Molecular Biomedicine, Institute Bio-science, Universiti Putra Malaysia, Serdang, Selangor, Malaysia. Cells were cultured in RPMI 1640 media containing 10% fetal bovine serum (FBS). Cell lines were incubated overnight at 37 °C in 5% CO_2_ for cell attachment. The cells were maintained by sub-culturing in 25 cm^2^ tissue culture flasks. Cells growing in the exponential phase were used for cell viability assay.

#### 3.9.2. MTT (3-(4,5-Dimethylthiazol-2-yl)-2,5-diphenyltetrazolium bromide) Assay

The assay was conducted as follows: Cancer cells were seeded in 96-well plates at a density of 1  ×  10^4^ cells/well in 100 μL of media. After 24 h, the medium was removed and the cells were incubated for 3 days in the presence and absence of various concentrations of sweet basil leaves extract [test extracts were prepared in 0.1% Dimethyl sulfoxide (DMSO) and serially diluted with media to obtain appropriate concentrations]. The following concentrations of extracts were used: 10–160 µg/mL. Cells in the control group received only media containing 0.1% DMSO. After incubation, the test compound containing media was removed and washed with 200 μL of PBS followed by addition of 20 μL of MTT reagent (5 mg/mL MTT in PBS) and incubated for 4 h at 37 °C. The medium was removed and 100 μL DMSO was added and the absorbance measured using a micro plate reader at 540 nm followed by the calculation of percentage viability. 0.1% (*v*/*v*) DMSO in medium was used as negative control. The cell viability was determined using the formula:

Viability (%) = 100 − (absorbance of cells treated with extract − absorbance of cells treated with 0.1% DMSO / absorbance of cells treated with 0.1% DMSO) × 100
(2)

Each point represents the mean of triplicate experiments.

### 3.10. Statistical Analysis

Statistical analysis was performed using Statistical Analysis System (SAS version 9.2, SAS Institute Inc., Cary, NC, USA). Mean separation test between treatments was performed using Duncan’s Multiple Range Test (ANOVA was performed before the Duncan’s Multiple Range test) and *p*-value of <0.05 was regarded as significant.

## 4. Conclusions 

To improve the nutritional quality and health benefits of herbal products, it is necessary both to devise an appropriate method and to identify the most suitable stage for implementation, which should be accomplished not only during the plantation and agronomy periods, but also post-harvest. From the present study, it can be concluded that UV-B irradiation with 3.60 W/m^2^ intensity and 8-h duration was the most effective in increasing the quantity of secondary metabolites in sweet basil leaves, leading to improvements in its pharmaceutical quality. UV-B irradiation led to the induction of cinnamic acid synthesis in sweet basil leaves, with this acid not being detected in non-treated plants. Enhancement of flavonoids by UV-B could be attributed to induction of CHS enzyme activity. UV-B treated leaves exhibited more potent antiproliferative activity against a breast cancer cell line (MCF-7) than did the non-treated plants. This demonstrates that the increase of phytochemicals via optimized UV-B irradiation conditions could also enhance bioactivity of the leaf extract, and might improve the potential application of nutraceutical products derived from sweet basil leaves. Antiproliferative and antioxidant activity of sweet basil mostly correlated with flavonoids followed by phenolic acids.

## Figures and Tables

**Table 1:** Effect of UV-B irradiation on TFC, TPC and individual flavonoid and phenolic acids content from sweet basil leaf extracts.

UV-B (W/m^2^)	TFC (mg QE/g DM)	TPC (mg GAE/g DM)	Gallic Acid (mg/g DM)	Cinnamic Acid (mg/g DM)	Ferulic Acid (mg/g DM)	Quercetin (mg/g DM)	Catechin (mg/g DM)	Kaempferol (mg/g DM)	Rutin (mg/g DM)	Luteolin (mg/g DM)
Control	22.26 ± 1.57 ^d^	48.22 ± 2.12 ^b^	4.76 ± 0.344 ^c^	ND	3.29 ± 0.209 ^c^	2.73 ± 0.183 ^c^	2.16 ± 0.156 ^d^	1.75 ± 0.155 ^d^	1.16 ± 0.112 ^c^	ND
2.30	37.18 ± 1.19 ^b^	50.41 ± 3.21 ^b^	5.22 ± 0.275 ^b^	3.24 ± 0.215 ^b^	4.10 ± 0.224 ^b^	3.48 ± 0.325 ^b^	3.50 ± 0.176 ^b^	2.44 ± 0.162 ^b^	1.52 ± 0.106 ^b^	0.88 ± 0.053 ^a^
3.60	41.25 ± 1.88 ^a^	56.12 ± 3.45 ^a^	6.46 ± 0.311 ^a^	3.91 ± 0.264 ^a^	4.88 ± 0.218 ^a^	3.87 ± 0.266 ^a^	4.00 ± 0.325 ^a^	2.90 ± 0.255 ^a^	1.96 ± 0.124 ^a^	0.86 ± 0.070 ^a^
4.80	27.62 ± 1.27 ^c^	49.16 ± 2.16 ^b^	4.11 ± 0.282 ^d^	3.08 ± 0.372 ^b^	ND	3.10 ± 0.304 ^b^	3.10 ± 0.224 ^c^	2.12 ± 0.183 ^c^	0.93 ± 0.067 ^d^	0.64 ± 0.046 ^b^

Data are means of triplicate measurements ± standard deviation. Means not sharing a common single letter in each column for each measurement were significantly different at *p* < 0.05. ND: not detected.

**Table 2:** Effect of UV-B irradiation time on TFC, TPC and individual flavonoid and phenolic acids content from sweet basil leaf extracts (UV-B: 3.60 W/m^2^).

Time (h)	TFC (mg QE/g DM)	TPC (mg GAE/g DM)	Gallic Acid (mg/g DM)	Cinnamic Acid (mg/g DM)	Ferulic Acid (mg/g DM)	Quercetin (mg/g DM)	Catechin (mg/g DM)	Kaempferol (mg/g DM)	Rutin (mg/g DM)	Luteolin (mg/g DM)
Control	20.88 ± 1.40 ^d^	44.52 ± 2.29 ^d^	4.32 ± 0.324 ^c^	ND	2.43 ± 0.144 ^d^	2.82 ± 0.142 ^c^	2.44 ± 0.165 ^e^	1.59 ± 0.140 ^e^	1.27 ± 0.138 ^b^	ND
4	37.76 ± 1.52 ^b^	47.53 ± 2.57 ^c^	4.59 ± 0.208 ^c^	3.41 ± 0.146 ^b^	3.16 ± 0.173 ^c^	3.24 ± 0.226 ^b^	3.86 ± 0.328 ^c^	2.74 ± 0.216 ^c^	1.50 ± 0.109 ^b^	0.70 ± 0.081 ^b^
6	40.11 ± 1.29 ^a^	51.12 ± 2.73 ^b^	5.12 ± 0.264 ^b^	3.96 ± 0.188 ^a^	4.55 ± 0.269 ^b^	3.45 ± 0.247 ^b^	4.24 ± 0.237 ^b^	3.18 ± 0.227 ^b^	1.53 ± 0.122 ^b^	0.93 ± 0.056 ^a^
8	41.19 ± 1.46 ^a^	55.79 ± 2.31 ^a^	6.07 ± 0.416 ^a^	3.24 ± 0.247 ^b^	5.10 ± 0.320 ^a^	3.85 ± 0.166 ^a^	4.67 ± 0.216 ^a^	3.80 ± 0.148 ^a^	2.12 ± 0.144 ^a^	0.96 ± 0.072 ^a^
10	28.11 ± 1.75 ^c^	43.20 ± 2.70 ^d^	3.72 ± 0.244 ^d^	3.11 ± 0.263 ^b^	2.10 ± 0.108 ^e^	2.43 ± 0.204 ^d^	2.80 ± 0.155 ^d^	2.26 ± 0.117 ^d^	1.00 ± 0.172 ^c^	0.65 ± 0.059 ^b^

Data are means of triplicate measurements ± standard deviation. Means not sharing a common single letter in each column for each measurement were significantly different at *p* < 0.05. ND: not detected.

**Table 3:** Correlations (R^2^) among human breast cancer cell proliferation, antioxidant activity and identified phytochemicals from sweet basil extracts.

Phytochemicals	Antiproliferative Activity	Antioxidant Activity
TFC	−0.9031 **	0.9100 **
TPC	−0.8940 **	0.8862 **
Gallic acid	−0.8225 *	0.8325 **
Cinnamic acid	−0.7433 *	0.8039 *
Ferulic acid	−0.8661 **	0.8590 **
Quercetin	−0.9327 **	0.9168 **
Catechin	−0.8266 *	0.8892 **
Kaempferol	−0.9240 **	0.9315 **
Rutin	−0.8590 **	0.8872 **
Luteolin	−0.8229 *	0.8126 *

* and ** are significant at *p* < 0.05 and 0.01 respectively.
